# Cost-effectiveness of procedure-less intragastric balloon therapy as substitute or complement to bariatric surgery

**DOI:** 10.1371/journal.pone.0254063

**Published:** 2021-07-28

**Authors:** Shweta Mital, Hai V. Nguyen

**Affiliations:** School of Pharmacy, Memorial University of Newfoundland, St. John’s, Canada; Queensland University of Technology, AUSTRALIA

## Abstract

**Background:**

Procedure-less intragastric balloon (PIGB) eliminates costs and risks of endoscopic placement/removal and involves lower risk of serious complications compared with bariatric surgery, albeit with lower weight loss. Given the vast unmet need for obesity treatment, an important question is whether PIGB treatment is cost-effective—either stand-alone or as a bridge to bariatric surgery.

**Methods:**

We developed a microsimulation model to compare the costs and effectiveness of six treatment strategies: PIGB, gastric bypass or sleeve gastrectomy as stand-alone treatments, PIGB as a bridge to gastric bypass or sleeve gastrectomy, and no treatment.

**Results:**

PIGB as a bridge to bariatric surgery is less costly and more effective than bariatric surgery alone as it helps to achieve a lower post-operative BMI. Of the six strategies, PIGB as a bridge to sleeve gastrectomy is the most cost-effective with an ICER of $3,781 per QALY gained. While PIGB alone is not cost-effective compared with bariatric surgery, it is cost-effective compared with no treatment with an ICER of $21,711 per QALY.

**Conclusions:**

PIGB can yield cost savings and improve health outcomes if used as a bridge to bariatric surgery and is cost-effective as a stand-alone treatment for patients lacking access or unwilling to undergo surgery.

## Introduction

Bariatric surgery is the most effective and cost-effective treatment for obesity compared with other obesity treatments [1–6]. However, access to bariatric surgery is extremely limited owing to financial and insurance constraints and shortage of bariatric surgeons; for instance, only 0.5% of eligible patients in the United States (US) have access to bariatric surgery each year [7].

Intragastric balloon (IGB) therapy–which involves placing gas- or saline-filled balloon inside the stomach—is an alternative procedure that can induce temporary weight loss [8]. This technique has recently gained popularity after the US Food and Drug Administration (FDA) approved two IGBs: Orbera^®^ (fluid-filled balloon) in 2015 and Obalon^®^ (gas-filled balloon) in 2016 [9]. In addition to being used as stand-alone treatment to achieve modest weight loss in patients with mild or moderate obesity, recent studies have examined use of IGBs as a potential bridge to bariatric surgery to achieve pre-operative weight loss [10].

The latest innovation in the field of IGBs is the Elipse™ balloon [11], which is unique in that it is the first procedure-less intragastric balloon (PIGB). It is currently being used in over 30 countries across Europe, Asia and Latin America [12] and the process for its pre-market approval by the US FDA is ongoing [13]. Unlike previous balloons, PIGB does not require endoscopy for either insertion or removal [11]. Consequently, it eliminates the costs and risks associated with endoscopy and sedation [14]. Further, weight loss effects of PIGB are similar to or higher than other FDA-approved IGBs [14].

PIGB also offers several advantages compared with bariatric surgery. First, as it is non-invasive, intervention costs of PIGB are lower than bariatric surgery [15]. Second, adverse events with PIGB are less likely and in most cases of a major complication, the balloon can be endoscopically removed [16]. Moreover, unlike bariatric surgery, existing studies of PIGB have not reported any mortality associated with the intervention [14, 16]. As with other IGBs, however, a key limitation of PIGB is that it generates lower weight loss than bariatric surgery. For instance, percentage of body weight lost on average with PIGB was 14% after 1 episode of treatment (lasting 4 months) [16] compared with 32% in 1–2 years after gastric bypass [17]. Furthermore, while long-term evidence on weight loss effects of PIGB is lacking, limited evidence (at 12 months after treatment initiation) suggests that patients regain weight after balloon removal [14].

Given the vast unmet need for obesity treatment and the unique advantages of PIGBs relative to other IGBs and bariatric surgery, albeit with lower weight loss than bariatric surgery, an important question for policymakers and clinicians is whether treatment with PIGB is cost-effective—either as stand-alone treatment or as bridge to bariatric surgery. To our knowledge, there is not yet evidence on the cost-effectiveness of PIGBs (and IGBs more generally) relative to bariatric surgery to shed light on this question. This study fills this knowledge gap by examining the cost-effectiveness of PIGB compared with the two most commonly performed bariatric surgeries (i.e., gastric bypass and sleeve gastrectomy) and no treatment among patients with morbid obesity. In addition to a direct comparison of cost-effectiveness of these treatments, we examine two hybrid strategies in which PIGB is offered as a first-line treatment prior to gastric bypass or sleeve gastrectomy.

## Methods

### Procedure-less intragastric balloons and their characteristics

The procedure-less intragastric balloon (Elipse™, Allurion Technologies, Natick, MA, USA) is delivered using a swallowable capsule [18]. Upon reaching the stomach, the balloon is filled with 550ml of fluid using a delivery catheter and the catheter is then withdrawn [14, 18]. The procedure is thus non-invasive and does not involve sedation; the position of the balloon is confirmed through an abdominal x-ray or fluoroscopy [18]. Within the stomach, the balloon works by occupying stomach capacity, inducing satiety and thereby reducing food intake [18]. The balloon stays in the stomach for 4 months after which a release valve opens and the balloon is excreted naturally [18].

### Treatment strategies

We estimated the cost-effectiveness of 6 strategies for weight loss. The first three strategies involved PIGB (‘PIGB -only, hereafter), gastric bypass (‘gastric bypass-only, hereafter) or sleeve gastrectomy (‘sleeve gastrectomy-only’, hereafter) as stand-alone treatment for all patients, respectively. In the next two strategies, PIGB was provided as first-line treatment to all patients. Patients who continued to suffer from morbid obesity after PIGB treatment underwent gastric bypass or sleeve gastrectomy thereafter (‘PIGB + gastric bypass’ and ‘PIGB + sleeve gastrectomy’, hereafter); those whose BMI fell below 35kg/m^2^ after PIGB treatment did not receive bariatric surgery immediately but did so once their BMI reached 35kg/m^2^ due to weight regain following PIGB treatment. Finally, the sixth strategy involved no weight loss treatment.

### Model structure and study cohort

We developed an individual patient-level Markov microsimulation model to compare the costs and quality-adjusted life years (QALYs) of the 6 strategies. This model allowed us to capture variation in weight loss effects across patients which in turn, influenced the timing of switch to bariatric surgery (if any) in the two hybrid strategies as described below. We simulated 10,000 adults aged 18–64 years with class 2 or class 3 obesity (i.e., BMI > = 35 kg/m^2^) and who had no contraindications for PIGB use [18]. These age and BMI ranges were chosen as (i) PIGB is recommended only for patients aged between 18 and 64 years [18]; and, (ii) bariatric surgery is primarily recommended for patients with BMI> = 35 kg/ m^2^ [19]. The proportion of patients with class 2 obesity (35< = BMI<40) versus class 3 obesity (BMI> = 40) was assumed to be 56% versus 44%, respectively, based on patterns of obesity prevalence among US adults [20].

**Fig 1:**
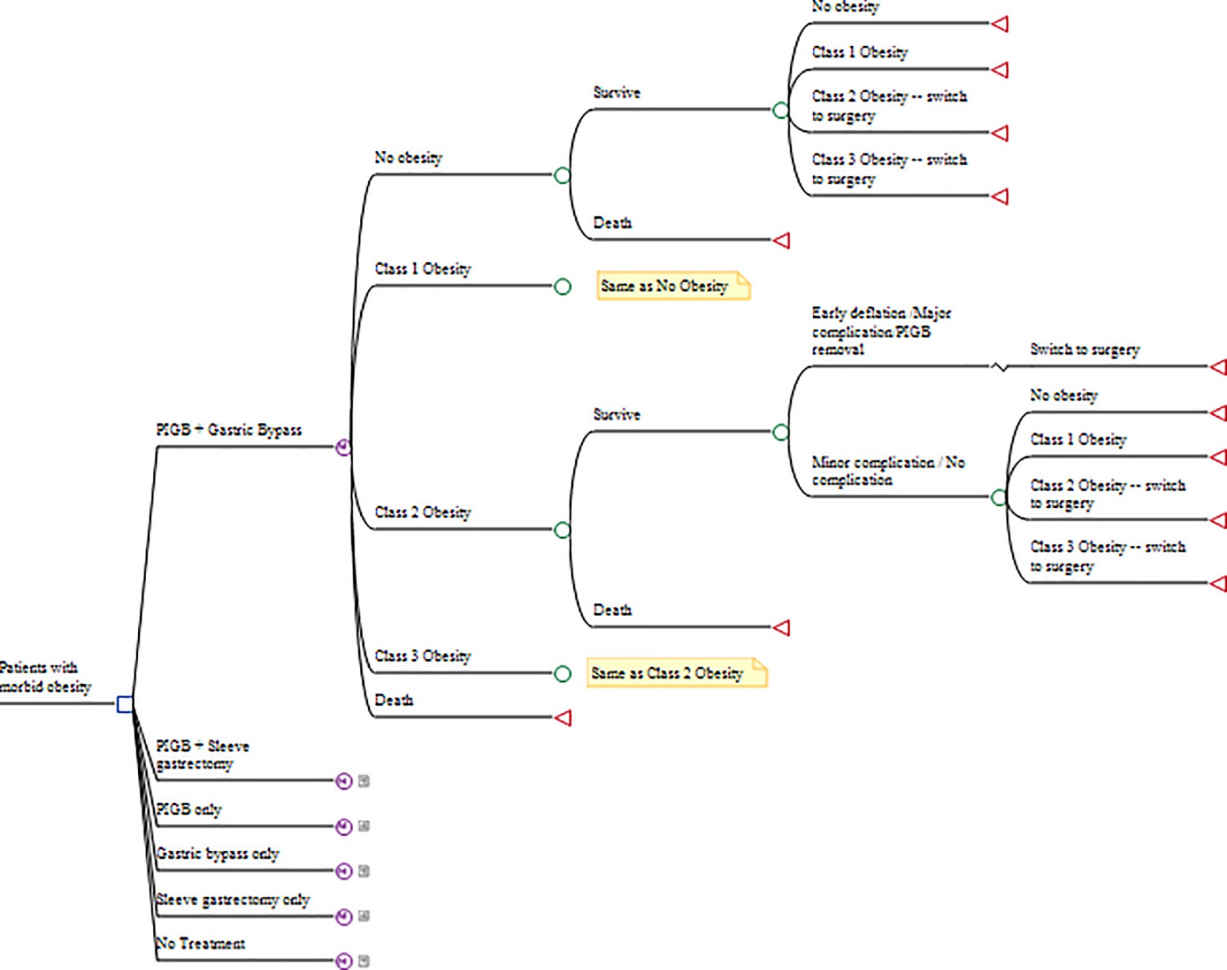
Simplified depiction of the Markov microsimulation model. Clinical pathways for PIGB + Sleeve gastrectomy and PIGB only strategies are the same as for PIGB + Gastric bypass, except that in PIGB only, patients do not switch to surgery if they are in Class 2 or Class 3 obesity health states. For patients switching to surgery after unsuccessful PIGB treatment, clinical pathways are the same as those for patients directly undergoing surgery. Clinical pathways after ‘Gastric bypass only’, ‘Sleeve gastrectomy only’ and ‘No treatment’ follow those previously published (Fig F1, Online supplementary materials in [21]).

The Markov microsimulation model comprised 5 health states: no obesity (BMI <30), class 1 obesity (30< = BMI<35), class 2 obesity (35< = BMI<40), class 3 obesity (BMI> = 40) and Death (**Fig 1**). Patients entering the model suffered from class 2 or class 3 obesity and underwent treatment (with PIGB, gastric bypass or sleeve gastrectomy depending on strategy) in the first cycle. After the first cycle, patients in the PIGB-only and gastric bypass/sleeve gastrectomy-only strategies transitioned across health states depending on extent of weight loss achieved by PIGB or surgery. Meanwhile, patients in the hybrid strategy who were still eligible for bariatric surgery (i.e., had BMI> = 35 kg/m^2^) underwent surgery. During the PIGB treatment, patients faced risk of major or minor complications. Major complications required balloon removal. Some patients could also experience early balloon deflation and expulsion. Patients in the hybrid strategy who experienced a major complication or early balloon expulsion underwent bariatric surgery in the next cycle. All patients undergoing bariatric surgery faced risk of surgery-related mortality as well as the risk of short and long-term complications. Complications were modelled as chance events for patients in a specific BMI health state, with an associated probability of occurrence. Patients who experienced these events incurred costs and disutility depending on type (major/minor) of complication. Complications after PIGB treatment and short-term complications after bariatric surgery could occur for patients with class 2 or class 3 obesity in the cycle that patients undergo treatment. Long-term complications after bariatric surgery could occur up to 5 years post-surgery, irrespective of the patient’s BMI health state.

We estimated costs from the health system perspective. Effectiveness was measured in terms of QALYs that captured both a patients’ length of life and their health-related quality of life (or utility). Cycle length was 4 months to match the length of an episode of PIGB treatment, and a lifetime horizon was used.

### Model inputs

Model inputs are presented in **Table 1** and detailed below.

**Table 1:** Model inputs.

Variable	Value	Distribution	Source
**Percent Total Weight Loss**			
PIGB (Month 4)	14.4% (4.9%) for class 2 obesity; 14.7% (4.2%) for class 3 obesity	Normal	[16]
Gastric Bypass (Month 120)	30.6% (7.7%)	Normal	[2]
Sleeve Gastrectomy (Month 120)	22.3% (5.6%)	Normal	[2]
**Mortality Hazard Rates**			
No obesity	1.83 (age 18–29); 0.72 (age 30–44); 1.08 (age 45–64); 0.89 (age >65)	*---*	[22]
Class 1 obesity	1.77 (age 18–29); 1.18 (age 30–44); 1.27 (age 45–64); 0.92 (age >65)	---	
Class 2 obesity	1.68 (age 18–29); 1.69 (age 30–44); 2.30 (age 45–64); 1.10 (age >65)	---	
Class 3 obesity	4.91 (age 18–29); 1.48 (age 30–44); 1.86 (age 45–64); 1.27 (age >65)	---	
**Probabilities**			
Proportion of patients with class 2 obesity	0.56	---	[20]
Proportion of patients with class 3 obesity	0.44	---	
Procedure related mortality (short term)			[23]
Gastric Bypass	0.0038 (9.5E-4)	Beta	
Sleeve Gastrectomy	0.0029 (7.25E-4)	Beta	
Procedure related mortality (long term)			
Gastric Bypass	0.0072 (0.0018)	Beta	
Sleeve Gastrectomy	0.0034 (8.5E-4)	Beta	
Early deflation	0.006 (0.002)	Beta	[16]
Major complication (short-term)			
PIGB	0.036 (0.009)	Beta	[16]
Gastric Bypass	0.094 (0.024)	Beta	[24]
Sleeve Gastrectomy	0.058 (0.015)	Beta	[24]
Minor complication (short-term)			
PIGB	0.0006 (1.5E-4)	Beta	[16]
Gastric Bypass	0.171 (0.043)	Beta	[24]
Sleeve Gastrectomy	0.074 (0.019)	Beta	[24]
Major complication (long-term)			
Gastric Bypass	0.151 (0.038)	Beta	[24]
Sleeve Gastrectomy	0.083 (0.021)	Beta	[24]
Minor complication (long-term)			
Gastric Bypass	0.109 (0.027)	Beta	[24]
Sleeve Gastrectomy	0.107 (0.027)	Beta	[24]
**Costs (in US$)**			
Intervention			
PIGB	5,647	Gamma	Authors’ calculation^a^
Gastric Bypass	30,235 (5,033)	Gamma	[2]
Sleeve Gastrectomy	26,328 (6,248)	Gamma	[2]
Follow up visits^b^			
Gastric Bypass	805, 483, 322	Gamma	[2, 25]
Sleeve Gastrectomy	805, 322, 161	Gamma	[2, 25]
Dietary supplements after bariatric surgery (annual)	100 (25)	Gamma	[2]
*Complications*			
Major complication (short-term)			
PIGB	2,695 (674)	Gamma	Assumption^c^
Gastric Bypass/Sleeve Gastrectomy	49,458 (12,364)	Gamma	[2]
Minor complication (short-term)			
PIGB	161 (40)	Gamma	Assumption^c^
Gastric Bypass/Sleeve Gastrectomy	1,517 (379)	Gamma	[2]
Major complication (long-term)			
Gastric Bypass/Sleeve Gastrectomy	54,454 (13,614)	Gamma	[2]
Minor complication (long-term)			
Gastric Bypass/Sleeve Gastrectomy	951 (238)	Gamma	[2]
*Health care costs (per year) by health state*:			
No obesity	4,152 (1,038)	Gamma	[2]
Class 1 obesity	4,881		
Class 2 obesity	5,744		
Class 3 obesity	6,997		
**Utilities**			
*BMI Specific Utilities*			
No obesity	0.91 (age 18–30); 0.89 (age 31–40); 0.86 (age 41–50); 0.83 (age 51–60); 0.81 (age 61–70); 0.79 (age > = 71)	Beta	[2]
Class 1 obesity	0.89 (age 18–30); 0.86 (age 31–40); 0.82 (age 41–50); 0.80 (age 51–60); 0.79 (age 61–70); 0.76 (age > = 71)		
Class 2 obesity	0.88 (age 18–30); 0.83 (age 31–40); 0.79 (age 41–50); 0.77 (age 51–60); 0.76 (age 61–70); 0.74 (age > = 71)		
Class 3 obesity	0.84 (age 18–30); 0.82 (age 31–40); 0.75 (age 41–50); 0.73 (age 51–60); 0.71 (age 61–70); 0.69 (age > = 71)		
*Disutility*			
Intervention related disutility			[3]
PIGB	0.002 (1.4E-4)	Beta	
Gastric Bypass/Sleeve Gastrectomy	0.025 (0.002)	Beta	
Major complication			
PIGB	0.014 (7.7E-4)	Beta	
Gastric Bypass/Sleeve Gastrectomy	0.042 (0.002)	Beta	
Minor complication			
PIGB	0.001 (7.2E-5)	Beta	
Gastric Bypass/Sleeve Gastrectomy	0.008 (5.8E-4)	Beta	

Values are Mean (SD). Standard deviations (SD) were obtained from the published literature where available. Where unavailable, SD was assumed equal to 25% of the mean value. Costs are measured in 2020 US dollars.

^a^Total cost of PIGB includes cost of balloon ($4,050 (SD: $1,012) calculated as £2,800 [15] converted to USD @ 1 GBP = 1.3897 USD as on January 18, 2018 [26] and adjusted for inflation), 6 physician visits (1 before balloon placement, 1 on day of balloon placement, 1 each in months 1–4) @ $161 (SD: $40) per visit [2], an abdominal x-ray to confirm balloon placement @ $97 (SD: $24) [27], one dose of aprepitant 125 mg (@ $90.73 per unit [28]) + Ondansetron (9 tablets @ $5.79 per unit [29]) + 2 doses of aprepitant 80 mg ($61.81 per unit [28]) + daily proton pump inhibitor starting 14 days before treatment (134 days x $2 per unit [30]) [16].

^b^Follow-up visits are based on the following schedule: 5 visits in year 1, 3 visits in year 2 and 2 visits per year beyond year 2 for gastric bypass and 5 visits in year 1, 2 visits in year 2 and 1 visit per year beyond year 2 for sleeve gastrectomy [25]. Each follow-up visit costs US$161 [2].

^c^ Cost of major complication with PIGB is assumed to be the weighted average of treatment with endoscopy costing $1,082 [31] and laparoscopy costing $26,328 (assumed equal to cost of laparoscopic sleeve gastrectomy procedure), where weights are based on proportion of complications treated with endoscopy vs. laparoscopy in Ienca et al. [16]. Cost of minor complication with PIGB is assumed to be the cost of one physician visit.

#### Weight loss effects

Weight loss effects at the end of 4 months of PIGB treatment were obtained from Ienca et al., a global multi-center study of 1,770 patients [16]. While several studies have examined weight loss effects of PIGB, we chose this study for two reasons: (i) it included a substantial western European patient population which would most closely resemble the US population; (ii) it reported weight loss following PIGB treatment for different BMI groups (<30, 30–40 and >40 kg/m^2^), allowing us to obtain weight loss effects specific to morbid obesity. However, Ienca et al. did not report weight loss or regain beyond treatment cessation at 4 months. Thus, beyond 4 months, we assumed that patients regained 7% of the initial weight lost during every 4-month period. This rate of weight regain was calculated based on meta-analytic estimates of weight change between PIGB treatment completion (at 4–6 months) and at 12 months [14]. For patients in the PIGB-only strategy, this weight regain of 7% every 4 months continued until the patient reached their initial weight. After that point, we assumed an annual BMI increase of 0.175kg/m^2^ which is similar to that for an average individual with obesity who does not undergo treatment [32]. Meanwhile, for patients in the hybrid strategies who achieved BMI<35 kg/m^2^ after PIGB treatment (and therefore, were not immediately eligible for bariatric surgery), this pattern of weight regain continued until their BMI reached 35 kg/m^2^ and were thus, eligible for surgery.

Weight loss effects for bariatric surgery were obtained from Alsumali et al., a recent cost-effectiveness analysis that presented long-term weight loss effects for gastric bypass (up to 10 years post-surgery) and sleeve gastrectomy (up to 8 years post-surgery) [2]. As only yearly weight loss effects were available for bariatric surgery, we linearly interpolated weight loss effects for each 4-month period to match the 4-month cycle length in our model. Beyond 10 years, we followed the literature in assuming that BMI remains constant at the level achieved in year 10 [2, 3].

#### Complications and mortality risks

Patients treated with PIGB could experience one of 3 types of complications during treatment: (i) early deflation and expulsion of balloon not requiring clinical intervention; (ii) major complications (such as balloon intolerance, small bowel obstruction, esophagitis, pancreatitis and gastric perforation) requiring endoscopic or laparoscopic removal of PIGB; and, (iii) minor complications (such as gastric dilation) [16]. Probabilities of these complications were obtained from Ienca et al. [16, 24]. Patients undergoing bariatric surgery faced the risk of short and long-term major and minor complications. Short-term complications could occur in the first 30 days while long-term complications could occur in years 1 to 5 post-surgery. We obtained the probability of these complications from a recent, high quality randomized controlled trial (RCT) [24].

Patients in all strategies faced risk of mortality specific to their age and BMI. We obtained age-specific risk of mortality from the latest available US life tables [33] and applied BMI-specific hazard ratios to it [22]. Patients undergoing gastric bypass and sleeve gastrectomy faced risk of surgery-related death up to 1 year post-surgery [23]. There was no risk of death associated with PIGB [16].

#### Costs

Costs of each strategy included cost of intervention and follow-up, general BMI-specific health care costs, and cost of treating complications (if any). Costs of PIGB included cost of the device, 6 physician visits (1 visit each pre-intervention, on the day of balloon placement and in each month during treatment), cost of an abdominal x-ray to confirm balloon placement and cost of medications [16]. Costs of bariatric surgery included cost of the surgical procedure, cost of follow-up visits (5 visits in year 1, 3 visits in year 2 and 2 visits year 3 onwards for gastric bypass and 5 visits in year 1, 2 visits in year 2 and 1 visit year 3 onwards for sleeve gastrectomy [25]) and cost of dietary supplementation. These costs, along with the general BMI-specific health care costs, were obtained from the published literature [2]. All costs were estimated in 2020 US dollars and discounted at 3.5% per year [34].

#### Utility

Utility values were age and BMI specific and were obtained from Alsumali *et al* which estimated EQ-5D scores based on data from the US Medical Expenditure Panel Survey [2]. In addition to the health state-specific utility, we applied utility decrements related to the intervention and its complications. Specifically, following existing literature, we assumed that bariatric surgery and its major complications resulted in utility decrement for 6 weeks while minor complications resulted in utility decrement for 4 weeks [3]. As PIGB is non-invasive and its complications are less severe than bariatric surgery, we assumed that utility decrement from balloon placement was half that of bariatric surgery and lasted only 1 week. Further, utility decrement from complications of PIGB was half that due to bariatric surgery and lasted 4 weeks for a major complication and 1 week for a minor complication. We varied these utility decrements in the one-way sensitivity analyses (described below). All utility values were discounted at 3.5% per year [34].

### Cost effectiveness analysis

We estimated the total costs and QALYs of the six strategies. We removed any strategies that were dominated in a simple sense (i.e., strategies that cost more but yielded fewer QALYs). We then estimated the Incremental Cost Effectiveness Ratio (ICER) as the ratio of the difference in total costs to the difference in total QALYs gained between two strategies and removed any strategies that were extended dominated (i.e., had a higher ICER than a more effective strategy). Among the remaining strategies, a strategy was considered cost-effective relative to another strategy if the ICER was below the conventional willingness-to-pay threshold of $100,000 per QALY.

We conducted several additional analyses. First, to address parameter uncertainty, we conducted conventional one-way sensitivity analyses in which we varied all costs and utilities in a range of ±25% of base case values [35], and probabilistic sensitivity analyses (PSA) in which we assigned distributions to input parameters and performed 1,000 Monte Carlo simulations. Second, we examined robustness of our results to changes in magnitude of 4-month weight loss of PIGB. In this analysis, we used meta-analytic estimates of weight loss after PIGB treatment from Vantanasiri et al. [14], which are slightly smaller than the estimates from Ienca et al. used in the base case analysis (i.e., 12.75% vs. 14.4%-14.7%). Third, while no deaths have been reported in PIGB studies, the FDA has recently alerted to the risk of mortality from other liquid-filled IGBs that was reported after the approval of those balloons [36]. Therefore, in this analysis, we considered the hypothetical possibility of a small mortality risk of 0.025% from PIGB similar to that observed for other balloons [37].

Fourth, we conducted additional sensitivity analyses to examine alternative long-term weight dynamics after PIGB and bariatric surgery. Long-term weight regain after PIGB treatment is not yet known. Therefore, in the first of these analyses, we varied the magnitude of weight regain after PIGB treatment between 0% (i.e., no weight regain) and 14% (twice that used in the base case analysis). In the second analysis, we assumed that long-term weight regain after PIGB treatment was similar to that of other saline-filled IGBs which require endoscopic placement/removal but work similarly to induce satiety to promote weight loss [8, 18, 37]. Specifically, we used 3-year meta-analytic estimates [38] for Orbera IGB (the only other saline-filled IGB that is approved by FDA) to estimate the percentage of weight lost that was regained between 1 and 3 years after treatment, and assumed linear weight regain at this rate from year 2 onwards (until the patient achieved their initial weight). Finally, we used long-term weight loss data for bariatric surgery from a recent, large, multi-center RCT which compared weight loss after gastric bypass and sleeve gastrectomy [24]. For gastric bypass, total percent weight loss at the end of 5 years in this trial was lower than weight loss at the end of 10 years reported in Alsumali et al. (27% vs. 31%). However, weight loss for sleeve gastrectomy was slightly higher (22.8% vs. 22.3%). All analyses were conducted using TreeAge Pro 2020 R2.1 [39].

## Results

### Base case analysis

**Table 2** presents the results of the base case cost-effectiveness analysis. There are three key findings. First, adding PIGB as a bridge to bariatric surgery is less costly and more effective than bariatric surgery alone (Panel A). Specifically, ‘PIGB + sleeve gastrectomy’ dominates sleeve gastrectomy only, and ‘PIGB + gastric bypass’ dominates gastric bypass only. This finding is explained by the fact that even though adding PIGB treatment increases upfront procedure costs, eventual weight loss is greater than without PIGB treatment which lowers downstream health care costs and improves quality of life.

**Table 2:** Incremental cost effectiveness results, base case.

Strategy	Cost ($)	Incremental Costs ($)	Effectiveness (QALYs)	Incremental Effectiveness (QALYs)	ICER ($/QALY)
**Panel A: All strategies**					
No treatment	125,387	-	14.35	-	-
PIGB only	127,760	2,373	14.46	0.11	Ext. dominated
PIGB + Sleeve Gastrectomy	133,801	6,041	16.57	2.12	2,855
Sleeve Gastrectomy only	135,987	2,187	16.11	-0.46	Dominated
PIGB + Gastric Bypass	142,974	9,173	16.62	0.05	195,693
Gastric Bypass only	143,732	758	16.38	-0.24	Dominated
**Panel B: Undominated strategies**					
No treatment	125,387	-	14.35	-	-
PIGB + Sleeve Gastrectomy	133,801	8,414	16.57	2.23	3,781
PIGB + Gastric Bypass	142,974	9,173	16.62	0.05	195,693
**Panel C: PIGB only vs. No treatment**					
No treatment	125,387	-	14.35	-	-
PIGB only	127,760	2,373	14.46	0.11	21,711

All costs are in 2020 US dollars ($). ICER = incremental cost-effectiveness ratio; PIGB = procedureless intragastric balloon; QALY = quality adjusted life year.

Second, among all six strategies, the ‘PIGB + sleeve gastrectomy’ is the most cost-effective strategy (Panel B). ‘PIGB + sleeve gastrectomy’ costs $8,414 more than no treatment ($133,801 vs. $125,387), but it also yields 2.23 additional QALYs. The resulting ICER is $3,781 per QALY gained which is much lower than the WTP threshold of $100,000 per QALY. Meanwhile, ‘PIGB + gastric bypass’ generates 0.05 additional QALYs compared with ‘PIGB + sleeve gastrectomy’. However, it is also more costly ($142,974 vs. $133,801) due to higher procedure costs and greater risk of complications with gastric bypass. As a result, ‘PIGB + gastric bypass’ is not cost effective relative to ‘PIGB + sleeve gastrectomy’ with an ICER of $195,693 per QALY that exceeds the WTP threshold of $100,000 per QALY.

Finally, Panel C shows that if only compared with no treatment, PIGB costs $2,373 more and generates 0.11 additional QALYs, generating an ICER of $21,711 per QALY gained. This ICER is lower than the WTP threshold of $100,000 per QALY, suggesting that PIGB treatment alone is cost-effective relative to no treatment.

### Sensitivity analysis

**Fig 2:**
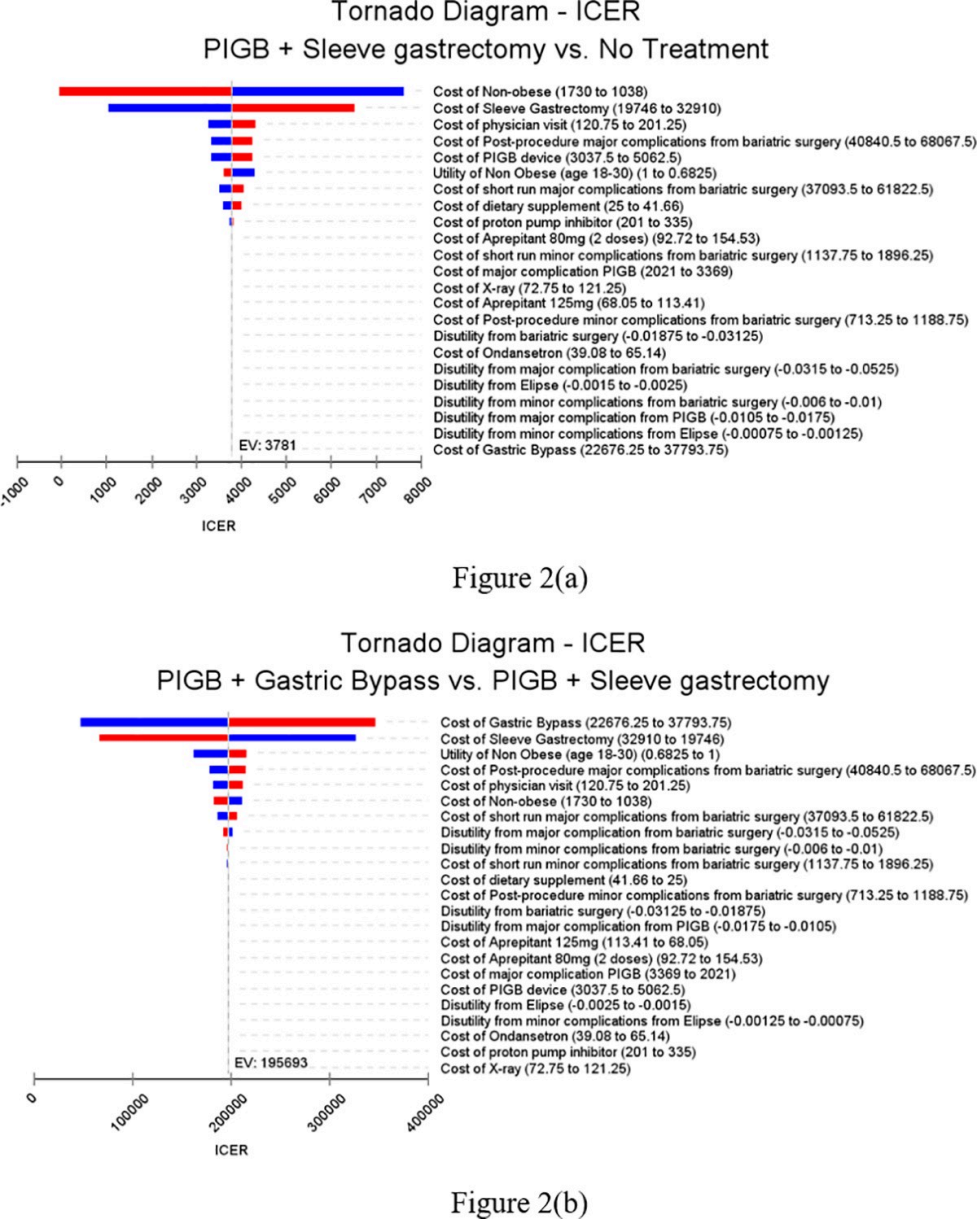
Tornado diagram. Fig 2(a) depicts the Tornado diagram for ‘PIGB + Sleeve gastrectomy’ versus no treatment while Fig 2(b) depicts that Tornado diagram for ‘PIGB + Gastric bypass’ versus ‘PIGB + Sleeve gastrectomy’.

Results of the one-way sensitivity analyses are presented in Tornado diagrams in **Fig 2**. **Fig 2(A)** shows that the ‘PIGB + sleeve gastrectomy’ strategy remained cost-effective or dominant relative to no treatment for all values of costs and utilities in the range of +/- 25% of base case values. **Fig 2(B)** shows that results for ‘PIGB + sleeve gastrectomy’ vs ‘PIGB + gastric bypass’ depend on procedure costs of gastric bypass and sleeve gastrectomy. If procedure cost of gastric bypass was lower or procedure cost of sleeve gastrectomy higher than that used in the base case analysis, the ‘PIGB + gastric bypass’ strategy would become cost-effective relative to ‘PIGB + sleeve gastrectomy’. However, for most values of these parameters and for all values of the remaining costs and utilities in the ranges considered, ‘PIGB + gastric bypass’ remained not cost-effective relative to the ‘PIGB + sleeve gastrectomy’.

**Fig 3:**
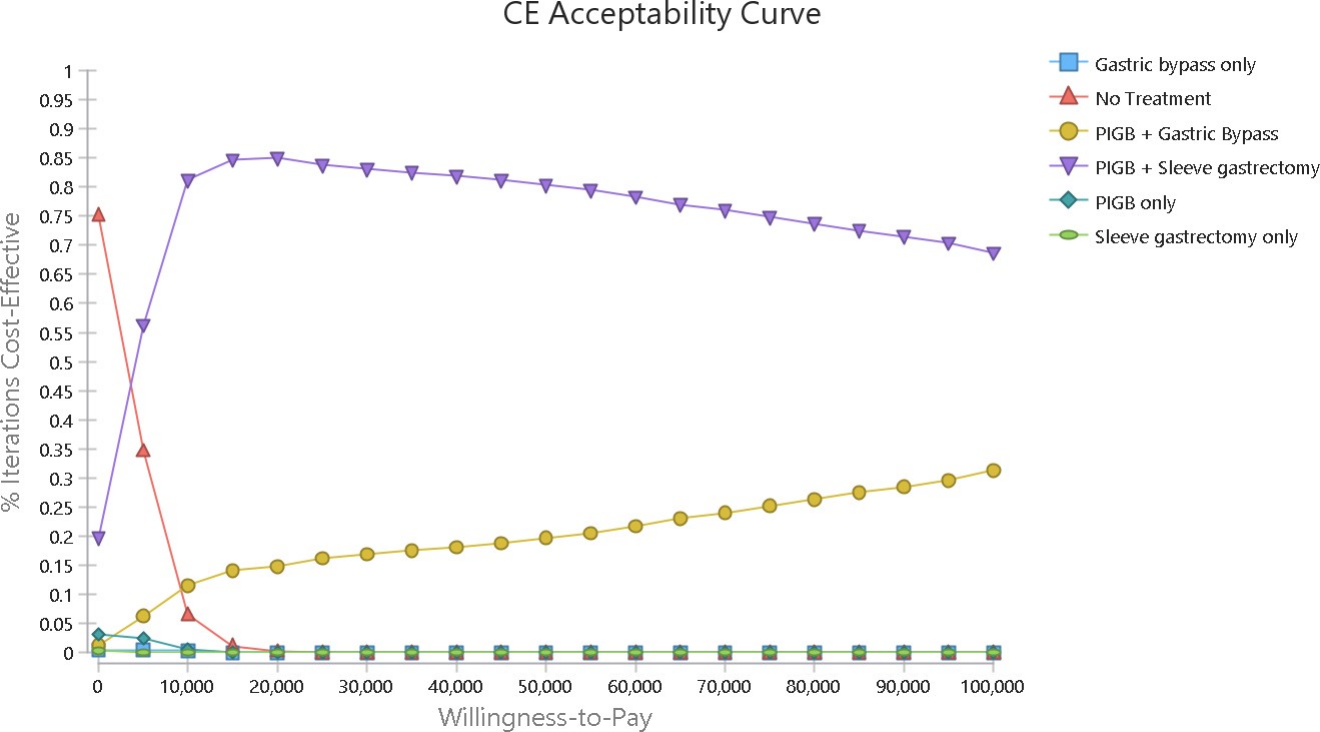
Cost-effectiveness acceptability curve.

Cost-effectiveness acceptability curves from the PSA indicate that at the WTP threshold of $100,000 per QALY, ‘PIGB + sleeve gastrectomy’ is cost-effective in 69% of iterations (**Fig 3**).

**Table 3** shows the results of the additional sensitivity analyses. We obtained similar results to the base case even when we used meta-analytic estimates for weight loss effects of PIGB instead of estimates from Ienca et al. (Panel A) and allowed for mortality due to PIGB (Panel B). ‘PIGB + sleeve gastrectomy’ also remained the most cost-effective treatment when we assumed that the extent of weight regain in the long-term after PIGB treatment was equal to that after Orbera treatment (Panel C), and when we used alternative data for weight loss effects for bariatric surgery (Panel D).

**Table 3:** Incremental cost effectiveness results, sensitivity analyses.

Strategy	Cost ($)	Incremental Costs ($)	Effectiveness (QALYs)	Incremental Effectiveness (QALYs)	ICER ($/QALY)
***Panel A*: *Meta-analytic estimates for weight loss from PIGB***					
No treatment	125,387	-	14.35	-	-
PIGB + Sleeve Gastrectomy	134,494	9,107	16.54	2.19	4,158
PIGB + Gastric Bypass	143,588	9,095	16.60	0.06	150,667
***Panel B*: *Allowance for PIGB -related death***					
No treatment	125,387	-	14.35	-	-
PIGB + Sleeve Gastrectomy	134,110	8,723	16.58	2.23	3,909
PIGB + Gastric Bypass	143,529	9,419	16.63	0.06	170,408
***Panel C*: *Alternative long-term weight loss effects for PIGB***					
No treatment	125,387	-	14.35	-	-
PIGB + Sleeve Gastrectomy	133,940	8,553	16.59	2.24	3,816
PIGB + Gastric Bypass	143,067	9,127	16.65	0.06	149,935
***Panel D*: *Alternative weight loss effects for bariatric surgery***					
No treatment	125,387	-	14.35	-	-
PIGB + Sleeve Gastrectomy	133,549	8,162	16.56	2.22	3,684
PIGB + Gastric Bypass	143,574	10,026	16.58	0.02	576,699

All costs are in 2020 US dollars ($). ICER = incremental cost-effectiveness ratio; PIGB = procedureless intragastric balloon; QALY = quality adjusted life year. Dominated strategies are excluded. In Panel A, total percent weight loss effects (Mean (SD)) for PIGB is 12.75% (3.2%) for patients in both Obese 2 and Obese 3 categories.

**Fig 4:**
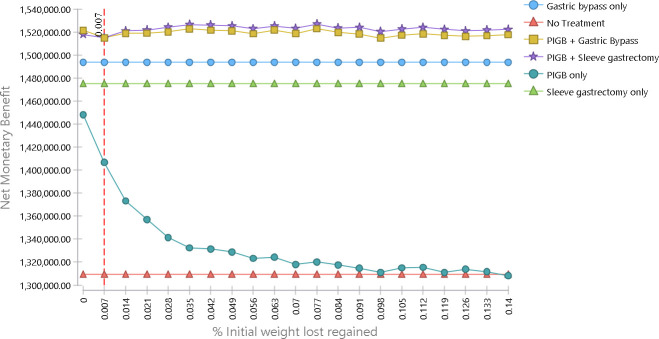
Net monetary benefit for different percentages of weight regain.

Further, when we varied the extent of weight regain after PIGB treatment between no weight regain and regain of 14% of weight loss per cycle, the ‘PIGB + sleeve gastrectomy’ was the most cost-effective unless weight regain was very small (smaller than 0.7% in each 4-month period; **Fig 4**). Overall, these sensitivity analyses indicate the robustness of our base case results.

## Discussion

In this study, we provided the first assessment of the incremental cost-effectiveness of PIGB as a substitute or complement to bariatric surgery. We found that using PIGB as an add-on treatment before bariatric surgery is both less costly and more effective than bariatric surgery alone. In particular, treatment with PIGB followed by sleeve gastrectomy is the most cost-effective. Furthermore, although PIGB alone is not cost effective versus bariatric surgery, it is a cost-effective treatment option compared with no treatment.

Our findings have several implications for policy and clinical practice. First, contrary to expectations that an add-on treatment to already expensive bariatric surgery would further increase health care costs, our results show that using PIGB as an add-on treatment *reduces* total costs and improves health outcomes compared with bariatric surgery alone. Consequently, as decision-makers look for ways to curb rising health care costs, it will be worthwhile to consider incorporating PIGB prior to bariatric surgery within the clinical care pathway.

Second, PIGB as a bridge therapy can be especially valuable for patients as it helps to achieve a lower BMI post-bariatric surgery. This is corroborated by findings from previous studies which suggest a positive correlation between pre-operative and post-operative weight loss [40]. Furthermore, intragastric balloon treatment can help allay fears and concerns of a more restrictive surgical procedure for some patients and ease their path towards bariatric surgery [41].

Third, even though weight loss effects of PIGB are modest and likely temporary, our results indicate that treatment with PIGB alone is still cost-effective for patients who lack access to bariatric surgery. Further, treatment with PIGB is non-invasive and reversible. Thus, it is likely to be of interest to patients who do not have bariatric surgery due to lack of insurance, fear of surgery-related risks or concerns over long-term weight regain after bariatric surgery [7].

Our study has a number of limitations. First, data on weight loss from PIGB specific to morbid obesity were available for a maximum duration of 4 months after treatment initiation. Beyond 4 months, we had to rely on estimates from a meta-analysis that also included patients with mild-and moderate obesity. However, we conducted sensitivity analyses to account for this data limitation, and our conclusions continued to hold. Second, our study relied on non-RCT data for weight loss effects of PIGB as RCT data was not available. In addition, no study has directly compared weight loss from PIGB with that from bariatric surgery so that weight loss effects for these treatments had to be obtained from separate studies. Future studies can re-examine the cost-effectiveness of PIGB vs bariatric surgery as longer-term weight loss data for PIGB from RCTs becomes available. Third, while our study highlights the economic value of PIGB as a bridge therapy to bariatric surgery, these findings are based exclusively on economic modelling; no clinical studies have examined this specific use of PIGBs prior to bariatric surgery. Thus, further clinical evidence on the use of PIGB prior to bariatric surgery will also be useful. Value of information analyses can be used to quantify the value of this additional research and the most efficient research design to collect such evidence [42]. Finally, there exists limited evidence that IGB therapy may result in gastric wall hypertrophy [43], which, in turn, could increase the risk of surgical leaks. While gastric wall changes have been found to be only transient [43] and previous clinical studies have demonstrated the feasibility of using IGB therapies as a bridge to bariatric surgery [10], future clinical studies can shed further light on the possibility of such risk.

In conclusion, findings from this study suggest that offering PIGB as a first-line treatment to all patients with morbid obesity prior to bariatric surgery yields cost savings and better health outcomes compared with bariatric surgery alone. Furthermore, for patients who lack access to or are unwilling to undergo bariatric surgery, treatment with PIGB alone is cost-effective compared with no treatment.
